# Splenic Marginal Zone Lymphoma in the Setting of Noncirrhotic Portal Hypertension

**DOI:** 10.1177/2324709615609385

**Published:** 2015-09-30

**Authors:** Saman Ratnayake, Ali Ammar, Rodd Rezvani, Greti Petersen

**Affiliations:** 1 Kern Medical Center, Bakersfield, CA, USA

**Keywords:** splenic marginal zone lymphoma, noncirrhotic portal hypertension, disseminated coccidioidomycosis

## Abstract

We present a case of a 65-year-old Hispanic man with a history of disseminated cutaneous coccidioidomycosis who presented to the emergency room for progressively worsening abdominal pain associated with shortness of breath. The patient was found to have pleural effusion and moderate ascites on physical examination. Abdominal ultrasound and computed tomography scan were consistent with moderate ascites and portal hypertension but negative for both liver cirrhosis and for venous or arterial thrombosis. Cytology of ascitic fluid was suggestive of portal hypertension and was negative for infection. Subsequent, thoracentesis was suggestive of exudative effusion and also negative for infection. Liver biopsy confirmed the absence of cirrhosis. Complete blood count indicated pancytopenia, whereas bone marrow biopsy and flow cytometry were suggestive of marginal zone lymphoma (MZL). Clinically, the patient’s shortness of breath was resolved by thoracentesis and paracentesis; however, his abdominal pain persisted. A diagnosis of idiopathic noncirrhotic portal hypertension in the setting of splenic MZL was made. The patient was transferred to a higher level of care for splenectomy; however, he missed multiple appointments. Since discharge, the patient has been seen in the outpatient setting and states that he is controlling his disease with diet and exercise; however, he continues to complain of intermittent shortness of breath with exertion.

## Introduction

Splenic marginal zone lymphoma (SMZL) is a very rare disease; it constitutes <1% of all non-Hodgkin’s lymphomas (NHLs).^1^ Although being an uncommon diagnosis, SMZL makes up about 25% of low-grade B cell neoplasms in patients who have undergone splenectomy.^2^ SMZL comprises a majority of chronic B cell leukemias and low-grade splenic lymphomas that do not fit the defining criteria of B cell CLL(Chronic Lymphocytic Leukemia), lymphoplasmacytic lymphoma, mantle cell lymphoma, follicular lymphoma, or hairy cell leukemia.^3^ The 2008 WHO classification system of lymphoid neoplasms divides them into 3 categories: (1) splenic marginal zone B cell lymphoma (±villous lymphocytes), (2) extranodal marginal zone B cell lymphoma of mucosa-associated lymphoid tissue (MALT) type (MALT-type lymphoma, MALT lymphoma), and (3) nodal marginal zone B cell lymphoma. The median age for MZL is 65 to 70 years, with the incidence in whites being twice that in other races.^1^

In Western countries, portal hypertension is typically the result of cirrhosis, with noncirrhotic portal hypertension accounting for less than 10% of cases. In addition, idiopathic noncirrhotic portal hypertension (IPH) is thought to account for 14% to 27% of all noncirrhotic portal hypertension cases.^4-6^ In this study, we present a patient with a history of disseminated cutaneous coccidioidomycosis, who presented with abdominal pain and shortness of breath and was subsequently diagnosed with SMZL and IPH on admission.

## Case Report

A 65-year-old Hispanic man with a significant past medical history of disseminated cutaneous coccidioidomycosis infection, hiatal hernia, and portal hypertensive gastropathy presented to the emergency room after a 5-day history of worsened shortness of breath associated with diffuse abdominal pain. The patient was in his normal state of health until approximately 5 months prior to admission when he began to notice a rash in the T4-T9 dermatome. At that time, the patient was diagnosed with disseminated cutaneous coccidioidomycosis. Over the course of the next 5 months, he began to experience abdominal pain and was seen in the outpatient clinic. Referral for abdominal ultrasound during this time demonstrated normal hepatic portal vein, hepatic artery, and IVC (Inferior Vena Cava) flow on color Doppler. A CT scan of the abdomen 1 month prior to admission showed moderate ascites, with a new-onset 2.02-cm nodule on the left lower lobe, left pleural effusion, splenomegaly, and ascites, and esophagogastroduodenoscopy showed grade 1 esophageal varices, diffuse portal hypertensive gastropathy, and a sliding hiatal hernia. Liver biopsy was subsequently done, which showed mild inflammatory activity and fibrosis.

On the day of admission, the patient’s vital signs were stable, and physical exam showed diminished breath sounds with rales bilaterally, abdominal distention with moderate fluid shift, caput medusa, umbilical protrusion, tenderness in all 4 quadrants, 15-cm liver span, splenomegaly, and a positive fluid wave. CBC(Complete Blood Count) displayed marked pancytopenia. The patient denied any history of alcohol abuse or recent travel.

**Figure 1.**
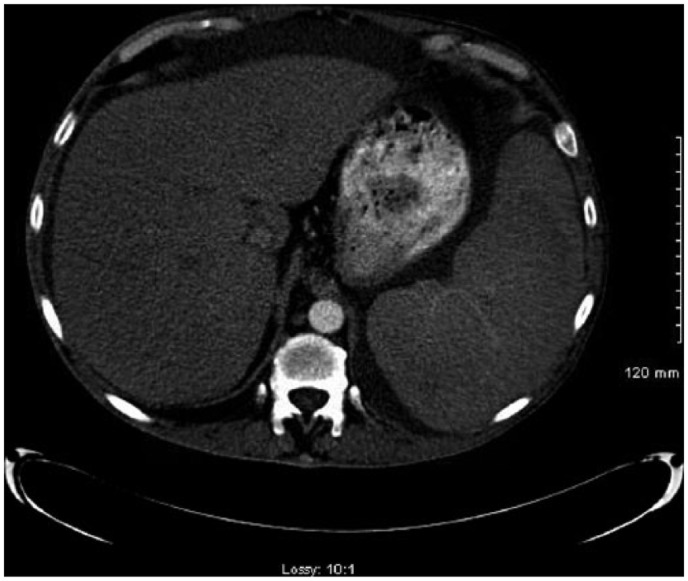
Computed tomography of abdomen, showing absence of fibrotic liver, which was consistent with the results of the liver biopsy.

**Figure 2.**
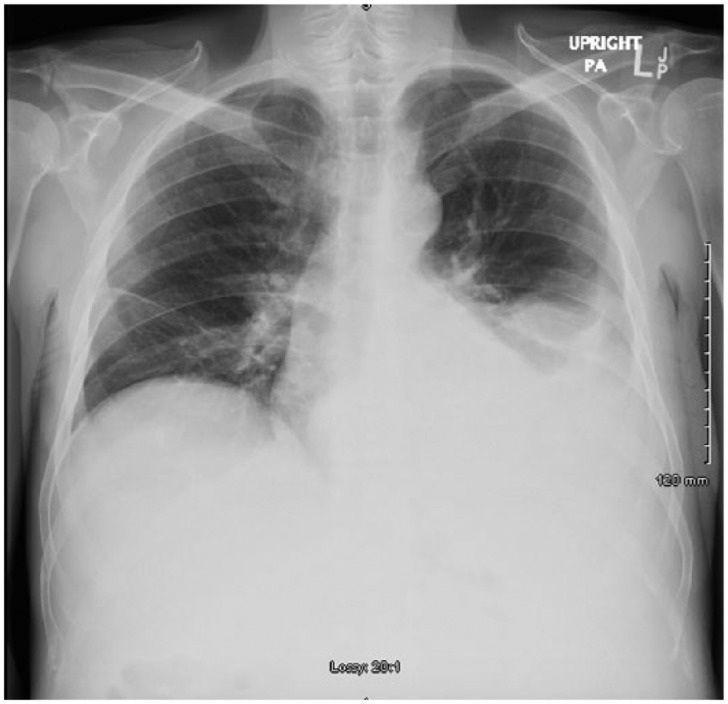
Left lower lobe effusion prior to thoracentesis.

**Figure 3.**
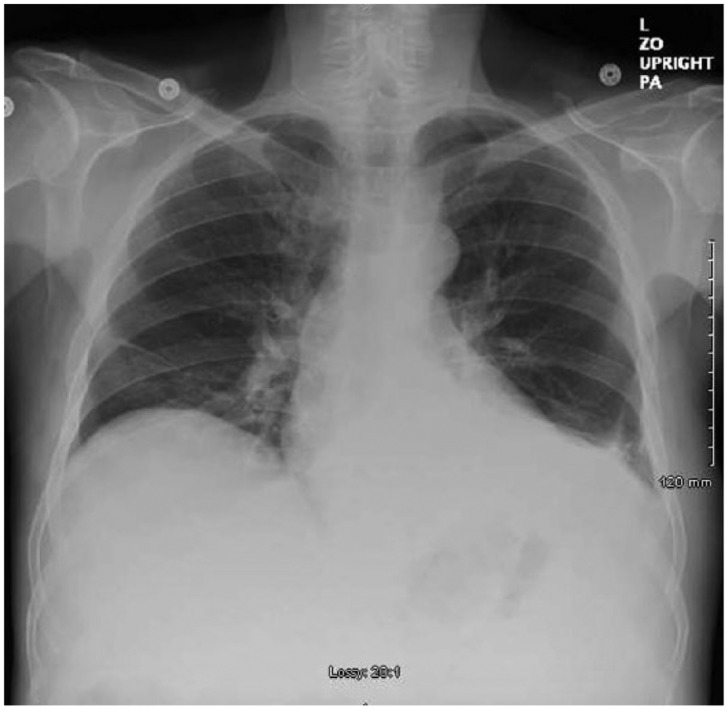
Resolved left lower lobe effusion status post thoracentesis; however, there was a new right middle lobe consolidation, which later resolved with a short course of antibiotic therapy.

**Figure 4.**
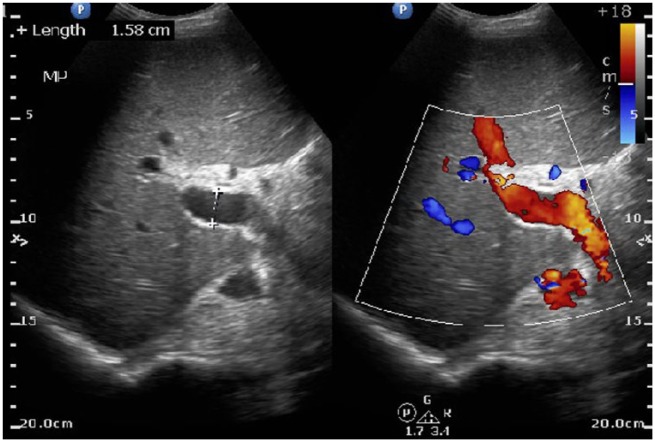
Ultrasound abdomen: ultrasound of liver identifies a patent portal vein, 158 mm in diameter, indicative of portal hypertension in this noncirrhotic patient.

On day 2, the patient continued to complain of abdominal pain and shortness of breath. Physical examination showed oxygen saturation of 88% with worsening ascites and still positive for rales. Repeat imaging of the abdomen was done on day 2 of admission, which confirmed ascites and pleural effusion that was previously reported (Figure 1). A thoracentesis and paracentesis helped relieve the patient’s symptoms (Figures 2 and 3). Pleural fluid was exudative. Paracentesis confirmed a SAAG(Serum Ascites Albumin Gradient) score of 1.2, suggesting portal hypertension. Microbiology of both body fluids showed no evidence of infection. CT and ultrasound of the abdomen were positive for ascites and splenomegaly but negative for cirrhosis. Considering thrombosis as a possible cause of portal hypertension, reevaluation of the ultrasound showed patent hepatic vasculature, including the portal vein, hepatic vein, and splenic vein (Figure 4). Laboratory results showed pancytopenia, elevated alkaline phosphatase levels, elevated GGT(Gamma-Glutamyl Transferase) levels, and elevated 5′ nucleotidase. Given the results, splenic causes of pancytopenia were considered.

On day 4, symptoms persisted. A peripheral blood smear showed pancytopenia and the presence of dacrocytes, elliptocytes, echinocytes, and anisocytosis. Hematology/Oncology was consulted and recommended flow cytometry of the bone marrow aspirate and a bone marrow biopsy. Both were completed and pending. On day 5, the patient complained of abdominal pain again, but denied shortness of breath and maintained an oxygen saturation above 94%. A therapeutic paracentesis was done, which relieved the abdominal pain.

**Figure 5.**
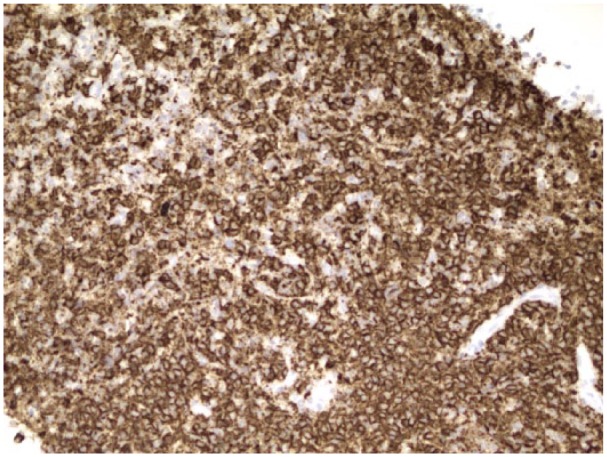
CD 20 on a fragment of tissue in the cell block: stain showing positive CD 20.

**Figure 6.**
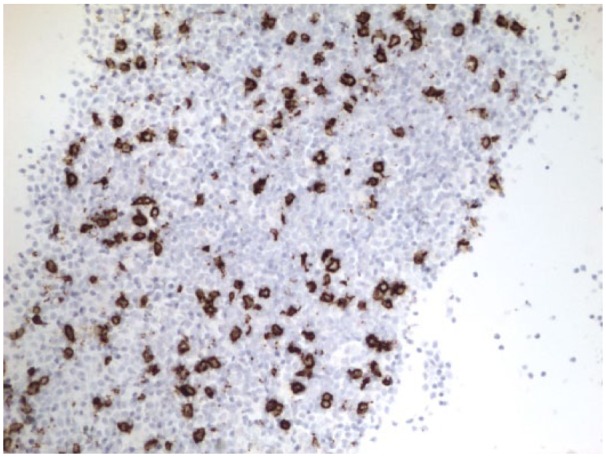
CD 20 on loose cells in the fluid that have aggregated: stain showing positive CD 20.

**Figure 7.**
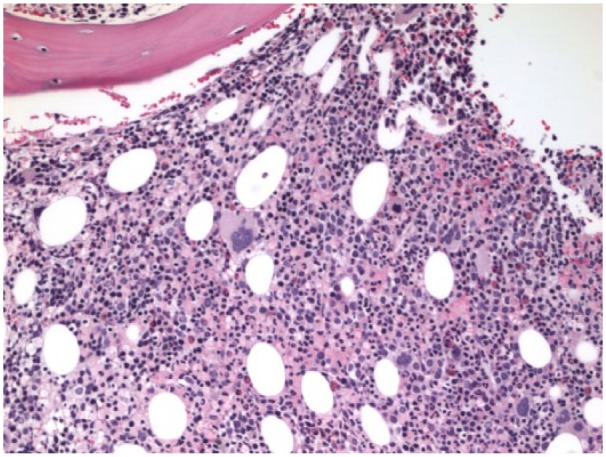
Bone marrow biopsy: showing hypercellular marrow with trilineage hyperplasia, mild eosinophilia, focal mild reticulin fibrosis, mild increase in iron and positive for a monoclonal B cell population. These findings were consistent with splenic marginal zone lymphoma.

Flow cytometry of the bone marrow showed a small population of monotypic B cells of uncertain clinical significance and T cells with minor immunophenotypic alterations but no abnormal myeloid cell populations. Furthermore, JAK-2 kinase was negative; B cells were positive for CD 20 and negative for CD 5, 10, and 11c (Figures 5 and 6). Subsequently, bone marrow biopsy showed a hypercellular marrow with trilineage hyperplasia, mild eosinophilia, focal mild reticulin fibrosis, and mild increase in iron and was positive for a monoclonal B cell population (Figure 7). These findings were consistent with SMZL. These results, in addition to the lack of hepatic congestion and/or cirrhosis, led to the diagnosis of noncirrhotic portal hypertension.

The patient no longer complained of shortness of breath throughout the rest of the hospitalization. Abdominal pain slowly returned; however, it remained stable and was controlled with acetaminophen with codeine. On day 14, the patient was discharged. To obtain a second opinion regarding hepatosplenomegaly and portal hypertension, the patient was referred to an outside gastroenterologist for a higher level of care. The patient was also advised to follow with his primary care physician within 4 weeks.

## Follow-up

The patient failed to follow up with his primary care physician and was seen in the outpatient clinic 3 months after discharge. Furthermore, he did not make an appointment with the referred gastroenterologist. On exam, the patient continued to be positive for hepatosplenomegaly. Repeat chest X-ray also did not show any pleural effusions but did display a new left-sided consolidation. The patient was recently contacted by telephone, and he denied any complaints of shortness of breath or abdominal pain. The patient attributed his lack of symptoms to his low salt diet and restriction of fluid intake as recommended in the clinic.

## Discussion

SMZL is a subset of MZL that occurs in the spleen and has been associated with being CD 19, 20, 22 positive and typically CD 5, 10, 43, 103, 25 negative.^7^ This is a slow-growing lymphoma that primarily presents with splenomegaly, hepatomegaly, and bone marrow involvement. Although CT is the traditional staging modality, a recent study has offered volume perfusion CT as an alternative method of staging and monitoring. Sauter et al,^8^ have shown that blood flow and volume are comparatively decreased in patients with splenic lymphoma. This has been presumed to be a result of sinusoidal compression from progressively increasing lymphoma cell population in the cords of the white pulp and infiltration of trabecular arteries. Although patients may be asymptomatic, treatment is considered when there is cytopenia, bulk mass, or development of symptoms. The therapy available for patients with SMZL is rituximab with or without adjunctive chemotherapy and splenectomy.

As discussed earlier, IPH is thought to account for 14% to 27% of cases of all noncirrhotic portal hypertension cases.^4-6^ Unlike portal hypertension caused by cirrhosis, the pressure gradient between the inferior vena cava and portal vein is within normal range <5 mm Hg in patients with IPH. The diagnosis of IPH is mainly clinical and is a diagnosis after exclusion of other causes of portal hypertension, including proving the patency of portal and hepatic veins.^9^ Patients may present with gastrointestinal bleeding from esophageal varices and/or left-upper-quadrant pain as a result of splenomegaly.^10^ Ascites may develop in 10% to 34% of cases.^9^ Common abnormal laboratory findings include anemia secondary to hypersplenism, followed by thrombocytopenia and leucopenia.^9^ In addition, as in the patient presented in this study, to rule out cirrhosis, a liver biopsy must be performed. Four pathological findings have been considered for diagnosis of IPH—hepatoportal sclerosis, periportal fibrosis, perisinusoidal fibrosis, and nodular regenerative hyperplasia.^11^ Treatment is aimed at controlling the 2 major symptoms: esophageal varices and splenomegaly. Endoscopic scleropathy and endoscopic variceal ligation are 2 techniques used to control variceal bleeding. It has been shown that endoscopic scleropathy and endoscopic variceal ligation are effective, with a recurrence rate of roughly 20%.^12^ Pancytopenia and severe hypersplenism are now indications for either splenectomy or partial splenic embolization.^10^

The use of rituximab ± splenectomy is standard for patients with symptomatic splenomegaly in SMZL.^13^ In this case, the patient did not receive rituximab, and although repeat CBCs were still indicative of pancytopenia, the patient has denied abdominal pain and shortness of breath at rest for the past 6 months. The patient failed to follow up for splenectomy at a tertiary center. Besides paracentesis, thoracentesis, and maintenance of low salt diet with fluid restriction, the patient’s lymphoma has not been treated properly because of lack of patient follow-up. More studies are needed to help identify a possible correlation between SMZL and IHP. In addition, further investigation is needed to help identify ideal treatment options based on stratification of disease severity.

There are many studies that describe cases of MZL; however, our study describes a case that is complicated by noncirrhotic portal hypertension in a patient with a history of disseminated cutaneous coccidioidomycosis. To our knowledge, most cases of lymphoma-associated portal hypertension are in the presence of splenic vein thrombosis or aneurysm. This case interestingly displayed a case of lymphoma-associated portal hypertension in the absence of splenic vein thrombosis or aneurysm. The patient had reaccumulating pleural and ascitic fluid and refused the typical chemotherapeutic regimen. However, symptoms have resolved.
